# Pin-tract myiasis after external bone fixation: A case report and review of literature

**DOI:** 10.1016/j.ijscr.2022.107247

**Published:** 2022-05-24

**Authors:** Nimesh Lageju, Durga Neupane, Lokesh Shekher Jaiswal, Upama Phuyal

**Affiliations:** a MBBS, B.P. Koirala Institute of Health Sciences, Dharan, Nepal; b Department of Surgery (Division of CTVS), B.P. Koirala Institute of Health Sciences, Dharan, Nepal; c The University of Texas at Arlington, TX, USA

**Keywords:** Pin-tract, Fracture, Debridement, Wound, Case report

## Abstract

**Introduction and importance:**

Myiasis has been reported as a complication of fracture treatment with external fixation. Therefore, physicians should be aware of the possible re-emergence of myiasis as a complication of surgery and the use of metal fixators.

**Clinical presentation:**

A 45-year-old male, non-diabetic, chronic alcoholic treated with external bone fixation and flap coverage for Gustilo-Anderson type IIIB comminuted fracture of shaft of left tibia and fibula with intact distal neuro-vascular system presented with multiple maggots with foul-smelling discharge from the pin tract. A diagnosis of abscess with pin-tract myiasis was made and managed with wound debridement and complete removal of maggots.

**Conclusion:**

We report this rare pin-tract complication to acknowledge how simple precautions, wound care, and avoidance of risk factors play a vital role in preventing such infestations. Therefore, physicians should be aware of the possible re-emergence of myiasis as a complication of surgery and the use of metal fixators.

## Highlights

• Pin-tract myiasis is a rare presentation of a neglected wound after external bone fixation.
• Myiasis is associated with poor hygiene which is still a public health problem in developing countries.
• Simple precautions, wound care, and avoidance of risk factors play a vital role in preventing this infestation.

## 1 Introduction

Infection is the most common and devastating complications of an open fracture, which has a reported incidence of 3—40% [1]. External fixation with metallic pins remains the standard orthopaedic treatment for open fractures, particularly those that are severe; however, this is associated with a variety of complications, including infection [2]. Most pin-site infections are bacterial in origin and are treated effectively by pin removal and wound care with or without antibiotics, but other infectious complications, although rare, have been reported [1], [2], [3]. Myiasis has been reported as a complication of fracture treatment with external fixation in an increasing number of cases recently [3], [4], [5]. Myiasis is a medical condition caused by Diptera larvae (maggots) in which a human being or other mammal is infected [6].

Herein, we report the case of pin-tract myiasis, a rare presentation of a neglected wound after external bone fixation, in a patient with a treated tibia and fibular fracture. The wound was treated with daily wound debridement and irrigation. Upon follow-up, the wound was healed and completely free of maggots. This work has been constructed in line of SCARE guidelines [7].

## 2 Case presentation

A 45-year-old-male presented to our emergency with an alleged history of a road traffic accidents resulting in pain and swelling of the left lower leg and a wound exposing the underlying bone. The wound was immediately washed with saline and packed with sterile gauges. An above-knee slab was applied. A diagnosis of Gustilo-Anderson type IIIB comminuted fracture of shaft of left tibia and fibula with intact distal neurovascular system was made. He was planned for external fixation and flap coverage. After 6 weeks, the pins were removed and the patient was discharged from our center. After that, the patient lost follow-up for almost 2 months.

**Fig. 1:**
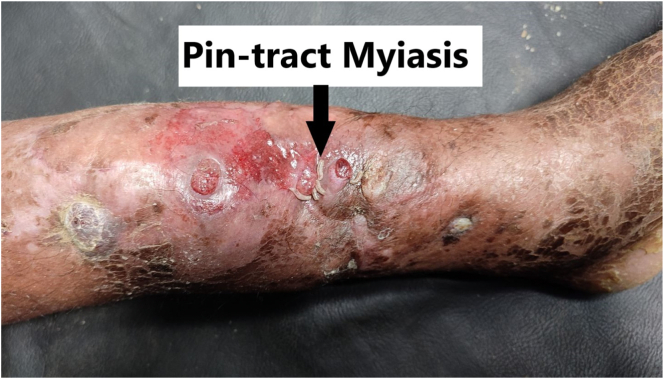
Pin-tract myiasis.

After 2 months, he presented to our center with complaints of swelling and foul-smelling discharge from the pin tract site along with maggots. A diagnosis of abscess with pin-tract myiasis (Fig. 1) was made. Wound culture was not done as it was unavailable in our hospital. Broad-spectrum antibiotics were started. It was managed with repeated daily debridement and irrigation of the wound. Upon close follow-up, the wound was healed and completely free of maggots. Psychiatric consultation and rehabilitation were done for alcoholism.

## 3 Discussion

With a reported incidence of 10% to 12.5%, pin site infection is one of the most common complications of external skeletal fixation for tibial fractures [8]. Several factors, including proper application of the fixation device with re-tightening of the pins, appropriate patient instruction, and close follow-up, have all contributed to a lower infection rate. The patient is given proper instructions for proper site care, such as cleaning with hydrogen peroxide twice a day. The patient has to follow up at the hospital every 2 weeks [3].

Traumatic myiasis is a parasitic infestation of tissue with fly larvae, also known as maggots, that happens when flies lay eggs in decomposing flesh or a suppurating wound [9]. Maggots can be either obligatory or facultative. Obligatory maggots are invasive and can infect living tissue, whereas facultative maggots prefer to infect dead or living hosts' necrotic tissue [10].

*Cochliomyia hominivorax* screwworm fly is the main species involved in wound myiasis in the New World. Wound myiasis is initiated when female flies oviposit on or near a wound. Upon hatching larvae, which have small spines on each body segment, penetrate headfirst into the tissues, burrow deeper perpendicular to the skin surface, and cause extensive destruction of tissue and a bloody discharge. *C*. *hominivorax* larvae differ from larvae of other fly species because they feed only on living flesh. The anatomic site around a lesion becomes swollen, and local tissue destruction can cause pain and secondary bacterial infection [11]. However, species identification was not done in our case.

There are only a few reports in the literature on pin-tract myiasis. For a failed fixation of a malleolar fracture, a 67-year-old diabetic woman was treated with an arthrodesis of the ankle joint with an external fixation. Similarly, two elderly diabetic men with trochanteric fractures of the femur were treated with external fixation. After failing to follow up for eight weeks, the patients developed myiasis along the external fixation pin holes. All of the patients were given wound debridement and lavage with hypertonic solutions, followed by povidone iodine local baths several times a day. A broad-spectrum antibiotic was also given intravenously. Within a week, the wounds were completely free of maggots [5].

Another case: A 32-year-old man with a history of alcohol and drug abuse (crack cocaine) was treated for a Gustilo type IIIA tibia fracture in his right leg with external fixation. He developed myiasis at pin sites three weeks later. The patient was given intravenous antibiotics and daily debridement and irrigation of the lesion to force the maggots out of the wound. Healing was visible after four days, with no signs of bacterial or parasitic infection [4].

Another case: For a cervical vertebrae fracture, a 39-year-old woman with a history of drug abuse was treated with a halo orthosis. After two weeks of discharge, she developed myiasis near the pin site. The wound was then debrided and irrigated, and the area was packed with a Clorpactin-soaked dressing. His wound appeared to be healing well after four weeks, with no signs of infection [3].

The external fixation device of our patient was properly placed and pins were properly tightened. Pin site care was also appropriate along with the prophylactic treatment with systemic antibiotics. However, poor post-discharge care of his wound and adjacent pin site, as well as a lack of close follow up likely lead to the development of myiasis in his pin site wound and surrounding skin. As a result, using an external fixation device in a patient who does not adhere to pin site care or follow-up will almost certainly result in such types of infection [3]. Myiasis is thought to have spread from the small infected wound next to the pin site in this case. Although the type of maggot involved in this case, whether facultative or obligatory, is unknown, the extent of involvement suggests that it is invasive. In order to treat myiasis effectively, the maggots must be completely removed, which was done in this case [12].

## 4 Conclusion

Diabetes, immobilization, drug and alcohol abuse, poor hygiene, low immune status, and lack of close follow-up are predisposing factors for the development of myiasis [3], [4], [5]. Alcohol abuse, poor hygiene, and lack of close follow-up were the potential risk factors in our patient. Despite the fact that bacterial infections are still the most common infectious complications in open fracture treatment, parasitic infections are becoming more frequent. Myiasis is associated with poor hygiene which is still a public health problem in developing countries. Although its incidence is low, this represents only the tip of the iceberg as a majority of the cases go undocumented. We report this rare pin-tract complication to acknowledge how simple precautions, wound care, and avoidance of aforementioned risk factors play a vital role in preventing such infestations. Therefore, physicians should be aware of the possible re-emergence of myiasis as a complication of surgery and the use of metal fixators.

## Consent for publication

Written informed consent was obtained from the patient for publication of this case report and accompanying images. A copy of the written consent is available for review by the Editor-in-Chief of this journal on request.

## Sources of funding

None.

## Ethical approval

The patient's consent was enough.

## Availability of data and materials

Upon request by Editor-in-chief. The data is available from the corresponding author.

## Research registration

No new surgical techniques or new equipment/technology was used.

## Guarantor

Nimesh Lageju.

Email: Nimesh.lageju@yahoo.com

## Provenance and peer review

Not commissioned, externally peer-reviewed.

## CRediT authorship contribution statement

NL, DN, LSJ, and UP contributed equally to the study concept, data collection, data analysis, preparation of this manuscript, and approval of its final version.

## Declaration of competing interest

None.
